# Development of a one-item version of the Orofacial Esthetic Scale

**DOI:** 10.1007/s00784-021-04049-6

**Published:** 2021-07-06

**Authors:** Stephanie Bela Andela, Ragna Lamprecht, Mike T. John, Swaha Pattanaik, Daniel R. Reissmann

**Affiliations:** 1 Department of Prosthetic Dentistry, Center for Dental and Oral Medicine, University Medical Center Hamburg-Eppendorf, Martinistr. 52, 20246 Hamburg, Germany; 2 Department of Diagnostic and Biological Sciences, School of Dentistry, University of Minnesota, Minneapolis, MN USA

**Keywords:** Orofacial appearance, Orofacial esthetics, Dental patient-reported outcome, Dental patient-reported outcome measures, Short instrument, Instrument development

## Abstract

**Objectives:**

Orofacial appearance is increasingly recognized as an important dental patient-reported outcome making instrument development and refinement efforts to measure the outcome better necessary. The aim of this study was to derive a one-item version of the Orofacial Esthetic Scale (OES).

**Materials and methods:**

OES data were collected from a consecutive sample of a total of 2113 adult English- or Spanish-speaking dental patients from HealthPartners dental clinic in Minnesota. Participants with missing data were excluded and analysis were performed using data from 2012 participants. Orofacial appearance was assessed with the English and the Spanish language version of the OES. Linear regression analysis was performed, with the OES item 8 (“Overall, how do you feel about the appearance of your face, your mouth, and your teeth?”) as the predictor variable and the OES summary score as the criterion variable, to calculate the adjusted coefficients of determination (R^2^).

**Results:**

The value of adjusted R^2^ was 0.83, indicating that the OES item 8 score explained about 83% of the variance of the OES summary score. The difference in R^2^ scores between the two language groups was negligible.

**Conclusion:**

The OES item 8 can be used for the one-item OES (OES-1). It is a psychometrically sound instrument for measuring orofacial appearance.

**Clinical relevance:**

Due to its easy application and sufficient psychometric properties, the OES-1 can be used effectively as an alternative to longer OES instruments in all areas of dental practice and research.

## Introduction

The patient perspective is fundamental for evidence-based dentistry [1, 2]. Orofacial appearance (OA) is increasingly considered to be one of the most important patient-reported outcomes (PROs) in dentistry. It is one of the main reasons for patients to pursue dental treatments [3], especially orthodontic treatments [4, 5]. Previous studies have recognized OA as one of the dimensions of oral health-related quality of life (OHRQoL) [6–8]. Furthermore, OA plays an important role in determining physical attractiveness of an individual [9] and impacts self-confidence and social interactions [10–13]. For instance, previous studies showed patients with misaligned teeth (malocclusion) and other dental abnormalities were perceived as less intelligent, less beautiful, and even socially disadvantaged [14–17]. As OA is an important PRO, a simple tool is needed to evaluate the impact of dental interventions on perceived OA. Furthermore, such a tool or instrument should also be applicable in large epidemiological studies. Accordingly, a short instrument is required minimizing burden for participants and simultaneously not adding too many items to the questionnaire battery, which might result in higher probability of missing data and non-response.

Currently, OA can be assessed by several instruments [18, 19], including the Oral Health Impact Profile (OHIP) [20], the most widely used multidimensional instrument for determining OHRQoL. OA can be assessed by various number of items ranging from six items of the original 49-item OHIP to one item for the 5-item short version [6, 7]. However, even the long OHIP does not seem to capture all aspects of OA [21]. Therefore, specific instruments to assess OA were developed. The Orofacial Esthetic Scale (OES) is a unidimensional 8-item instrument with sufficient validity and reliability, initially developed to comprehensively assess OA in prosthodontic patients [22, 23]. It has been translated and validated in several languages, and normative values for the general population are provided [24–30]. Although OES-8 is not a lengthy instrument and does not substantially burden respondents, yet a one-item questionnaire will further reduce costs and burden and expand its application to several settings. A one-item OES could increase research efficiency, facilitate application in a larger study framework, and increase patient compliance.

Such an approach using a global rating has appeal. That is, patients can consider all relevant aspects of the construct to be measured, can ignore aspects that are not relevant to them, and can differently weight the impact of relevant aspects according to their perception when responding to the global rating [31]. Single, global questions have in fact long been used in population surveys to measure general health status, quality of life (QoL), and health-related quality of life (HRQoL) [32–34]. The development of short versions is also a current trend for dental patient-reported outcome measures (dPROMs). One example in dentistry is the 5-item OHIP (OHIP-5) that is already validated in German [35], English [36], and Spanish [37]. This short version collects about 90% of the information from the original 49-item version, demonstrating its capabilities as a valuable tool for evaluating OHRQoL [35]. OHIP-5 provides the basis for the assumption that short versions are able to achieve comparable valid and reliable results despite reduction of the item pool. As an alternative, a single item dPROM would be even more promising.

Therefore, this study aimed to develop and validate a one-item OES.

## Methods

### Subjects, study design, and setting

In this cross-sectional study, a sample of 2113 English- and Spanish-speaking adult dental patients who had scheduled a dental appointment at HealthPartners dental clinics in MN, USA, were approached to participate in the study and were consecutively recruited from July 2014 to April 2016. From the initial sample, only patients with sufficient information to characterize the construct were included in the final analysis (*N* = 2012).

Questionnaires were sent to participants, who filled in the questionnaire at home and sent it back to the HealthPartners Institute. For more information on sampling, see also Reissmann et al. [29] and Simancas-Pallares et al*.* [30].

This research was conducted in accordance with accepted ethical standards for human-subject research practice, undergoing review and approval by the Institutional Review Board of the HealthPartners Institute in Minneapolis, MN (registration number A11-136). Informed consent was obtained from all participants prior to their enrollment.

### The Orofacial Esthetic Scale

Details of the development of OES have been published elsewhere [22, 23] and are briefly summarized here. We applied the English and the Spanish-language OES versions that were previously validated in the population of the current study [29, 30]. The OES consists of seven items addressing patients’ perceptions of specific esthetic components (i.e., appearance of face, mouth, teeth, and tooth replacement) and one item for the overall impression (item 8: “Overall, how do you feel about the appearance of your face, your mouth, and your teeth?”). The response format used is an 11-point rating scale, ranging from 0 (“very dissatisfied”) to 10 (“very satisfied”). Scores of items 1 through 7 can be summed up to form an OES summary score that can range from 0 through 70 points, with higher scores representing better esthetics. Since item 8 represents an overall impression and no specific esthetic component, it was deemed a candidate to serve as one-item OES.

### Data analyses

Participants’ socio-demographic characteristics and OES item and summary scores are presented using measures for central tendency (means) and variability (standard deviation; SD) for continuous measures, and frequencies and proportions for categorical measures. To ensure both language subgroups (English and Spanish) were comparable and could be analyzed collectively as one sample, subgroup characteristics were compared to test for statistically significant differences using two-sample t-test for continuous data (age and OES scores) and chi-squared test for categorical data (gender).

Linear regression analysis was performed to assess whether OES item 8 can be used for the one-item OES (OES-1). OES item 8 was used as the predictor variable and the OES summary score was used as the criterion variable for the linear regression model. Adjusted R^2^ was interpreted in terms of how much variance in OES summary score could be explained by OES item 8. Since there is no commonly accepted guideline for judgment of R^2^ values available, and an acceptable level depends on the research context, for this study, we considered values of at least 0.75 as satisfactory. As additional sensitivity analysis, regression models were calculated for each language subgroup separately.

All eight OES items were complete in 1931 (91.3%) participants. Only 595 missing answers were observed in 184 participants. For participants with one item with missing information in OES items 1 through 7, scores for these items were replaced by the median of the remaining items within a participant containing sufficient information. All participants with more than one item with missing information in OES items 1 through 7 and all participants with missing answers for item 8 were excluded from analyses.

All statistical analyses were performed using the statistical software package STATA/MP (Stata Statistical Software: Release 14.2. StataCorp LP, College Station, TX), with the probability threshold of a type I error set at 0.05.

## Results

### Characteristics of participants

Participants were on average 54.5 years of age (Table 1). However, participants from the Spanish-speaking subgroup were on average about 14 years younger than those from the English-speaking subgroup (*p* < 0.001). Slightly more than half of the participants (60%) were female with no statistically significant difference between groups (*p* = 0.834).

**Table 1:** Socio-demographic characteristics and OES scores for all participants and stratified by language-subgroups with statistical significance of between-group differences

	All	English	Spanish	Statistical significance
	*n* = 2012	*n* = 1702	*n* = 310	
	Mean (SD) or *n* (%)			*P-*value
Socio-demographic characteristics				
Age (years)	54.5 (16.1)	56.7 (15.8)	42.6 (12.1)	< 0.001
Gender (woman)	1205 (59.9)	1021 (60.0)	184 (59.4)	0.834
OES scores				
Item 1 (face)	7.7 (2.7)	7.7 (2.7)	7.6 (3.1)	0.618
Item 2 (profile)	7.7 (2.7)	7.8 (2.7)	7.5 (3.2)	0.078
Item 3 (mouth)	6.9 (3.1)	6.9 (3.1)	6.9 (3.3)	0.674
Item 4 (tooth alignment)	6.6 (3.3)	6.6 (3.2)	6.5 (3.5)	0.535
Item 5 (tooth shape)	6.8 (3.0)	6.9 (3.0)	6.8 (3.3)	0.591
Item 6 (tooth color)	5.9 (3.1)	5.9 (3.0)	6.1 (3.2)	0.253
Item 7 (gingiva)	7.2 (3.0)	7.2 (2.9)	7.1 (3.3)	0.507
Summary score	48.8 (18.2)	48.9 (17.8)	48.5 (20.1)	0.700
Item 8 (overall impression)	7.0 (2.9)	7.0 (2.8)	7.3 (3.0)	0.095

Mean OES scores for the individual items 1 to 7 in all participants ranged from 5.9 (item 6 — tooth color) to 7.7 points (item 1 — face, item 2 — profile; Table 1). Mean OES summary score was 48.8 and item 8 mean was 7.0 points. There were no statistically significant differences between the two language groups with regard to the individual item scores and the mean scores of OES (all *p* > 0.05).

### OES item 8 (OES-1) as predictor for OES summary score

The regression coefficient of 5.8 in the linear regression analysis indicates a 1-point difference of the OES item 8 score was related with a difference in OES summary score of 5.8 points (Table 2). The adjusted R^2^ of 0.83 in the model with all participants suggested that about 83% of variance in OES summary scores could be explained by OES item 8 score. The sensitivity analysis did not reveal any substantial differences in findings between the language groups with respect to regression coefficient and adjusted R^2^ (Table 2).

**Table 2:** Linear regression models characterizing the relationship between OES item 8 and OES summary score in all participants and in language subgroups

Sample	Predictor variable	Criterion variable	Coefficient	95% CI	P-value	Adj. R^2^
All participants						
	OES item 8	OES summary score	5.8	5.7–5.9	< 0.001	0.83
English-language participants						
	OES item 8	OES summary score	5.8	5.6–5.9	< 0.001	0.84
Spanish-language participants						
	OES item 8	OES summary score	6.1	5.8–6.4	< 0.001	0.82

## Discussion

This is the first study to develop and validate a one-item OES. The findings of this study indicate that OES-1 is a valid instrument to assess perceived OA.

Our results suggest that OES-1 sufficiently captures the OA construct even when compared across two language groups. The “overall impression of OA” item is a relevant and comprehensive indicator to measure OA in general and can replace the seven items of OES to produce a robust single-item scale. The R^2^ value indicates a “good model fit” as well as the ability of OES-1 to assess about 83% of the information of the original 8-item version, proving its suitability for accurately predicting perceived OA in general with good accuracy.

Our findings are comparable with those of other studies that aimed at developing shorter versions of dPROMs. However, several studies only performed correlation analyses and reported *r*-values. For example, a study on dental anxiety indicated that the scores of a single item were highly correlated with the original summary score (*r* = 0.77) [38]. Furthermore, the Swedish 14-item and 5-item short form of the OHIP (OHIP-S14 and OHIP-S5) correlated highly with the 49-item OHIP (*r* ≥ 0.97 for OHIP-S14, *r* ≥ 0.92 for OHIP-S5) [39, 40]. Also, the Spanish language 5-item OHIP (OHIP-Sp5) summary scores showed “very large” effect (*r* = 0.95) in correlation with OHIP-Sp14 as well as with OHIP-Sp49 [37]. Nevertheless, in many cases, the coefficient of determination R^2^ is simply the square of the correlation coefficient *r*, i.e., *r* = 0.95 would correspond to R^2^ = 0.90, demonstrating our findings do not differ substantially from those of Swedish and Spanish OHIP short forms. One study on German version of OHIP utilizing the same statistical methodology as in our study proved successfully that a 5-item version performed well in predicting summary scores of long OHIP version even in two different populations [35]. Adjusted R^2^ was 0.88 for the general population participants and 0.82 for temporomandibular disorders (TMD) patients, which is not too different from our findings. In contrast, a longer OHIP short form with 14 questions accounted for 94% of variance of the 49-item version [41]. German OHIP short forms with 14 items [42, 43] and with 21 items [44] explained 91% and 96% of the variance of the 49-item version, respectively. But it is not surprising that instruments with more items cover more information of the original instrument than shorter versions. Therefore, these findings do not question our findings. Accordingly, OES-1 performed similar to other abbreviated dPROMs.

Our study has several strengths. Most importantly, the study investigated regular dental patients — a highly relevant target population for the assessment of OA — using a large sample (n = 2012) ensuring sufficient statistical power and precise estimations. The diversity of the population ensures high generalisability of findings and facilitates implementation in most research settings. Moreover, OES-1 is derived from the existing OES-8, which has already proven itself in terms of validity and reliability [22]. We used a commonly applied method for imputation of missing data whilst taking into account that a person median imputation leads to the most accurate recovery and lowest bias across most conditions [45]. Furthermore, two different language versions of OES were used in the analysis. A recent study pooling OES data from English- and Spanish-speaking patients supported absence of differential item functioning across the two language versions [46]. Accordingly, this study suggests equivalence between the two language versions for OES-1 in terms of intercultural use. This increases comparability of the instrument with prospects for successful transferability to other language versions of OES-1. Possible limitations of our study are missing information on income, place of residence, or educational status, since it is known that socioeconomic differences potentially affect patient reported OA [47, 48]. Nevertheless, we assume that the large sample size and the inclusion of the entire population from various social strata have eliminated this potential drawback.

OES-1 is a very short and easy-to-apply questionnaire, suitable to measure the entire construct of OA in general and dental patient populations when time and resources are limited. Its brevity and easy application make the one-item OES a pragmatic and timesaving tool for large epidemiological studies, national health surveys, or routine dental practice where a multi-item OES questionnaire is not feasible. Historically, multi-item instruments were used more commonly and single-item instruments in comparison were assumed to be less reliable and valid, and more limited. Today, researchers advocate the use of single-item measures especially in clinical settings as they are less time consuming, reduce respondent burden, and costs of data collection [49]. These benefits make single-item measures easily applicable in non-clinical community settings such as mobile dental clinics. Even though multi-item instruments are better suited for complex constructs and can be more discriminating, they are also time-taking and can possibly lead to response errors [50].

The current study findings show that OES-1 is a practical, concise, and highly reliable measure of OA. Having said that, further testing of its validity and reliability in different settings and populations is needed. OES-1 represents a promising alternative to OES-8 and its convenient application will encourage increased application among dental researchers and providers. It can be used as a standardized global measure to monitor and evaluate treatment effectiveness. This enables dentists and patients to benefit from the results in the shortest possible time and engage in shared treatment decision-making. Having two OES instruments with different lengths would enable the measurement of OA in almost all research and clinical settings. The ease of use will also help with medical and dental interprofessional collaboration. A one-item dPROM such as OES-1 can contribute to effective communications with patients about expected treatment results [51]. It also furthers value-based oral health care [52] as it expedites the data collection and sharing process, leaving more time for dental care teams to engage in reflective learning and patient education. It can easily be used in resource limited settings and within large-scale surveys to allow cross-country comparisons.

The study demonstrated that OES-1 adequately captures the construct of OA. This provides a conceptually appealing and pragmatic opportunity that can be used in large epidemiological studies as well as in clinical trials and routine dental care. OES-1 paves way for other concise patient-centered outcome measures, which would help advance evidence-based dental practice.
